# Supplementary material for: Discovery of New 1,4,6-Trisubstituted-1H-pyrazolo[3,4-b]pyridines with Anti-Tumor Efficacy in Mouse Model of Breast Cancer
Source: Pharmaceutics. 2023 Feb 27;15(3):787. doi: 10.3390/pharmaceutics15030787 (PMC10057642; doi:10.3390/pharmaceutics15030787)
Supplement: Supplementary file 1 [file pharmaceutics-15-00787-s001.zip › pharmaceutics-2221318-supplementary.pdf]

Supplementary Information

Discovery of new 1,4,6-trisubstituted-1*H*-pyrazolo[3,4-*b*]pyridines with anti-tumor efficacy in mouse model of breast cancer

Maria Georgiou[1], Nikolaos Lougiakis[1], Roxane Tenta[2], Katerina Gioti[2], Stavroula Baritaki[3], Lydia-Evangelia Gkaralea[4], Elisavet Deligianni[3,4], Panagiotis Marakos[1*], Nicole Pouli[1], Dimitris Stellas[4*]

[1] Division of Pharmaceutical Chemistry, Department of Pharmacy, School of Health Sciences, National and Kapodistrian University of Athens, Panepistimiopolis Zografou, 15771, Athens, Greece

[2] Department of Nutrition & Dietetics, School of Health Sciences and Education, Harokopio University, Athens 17671, Greece

[3] Laboratory of Experimental Oncology, Division of Surgery, School of Medicine, University of Crete, GR-71003 Heraklion, Greece.

[4] Institute of Chemical Biology, National Hellenic Research Foundation, Athens, Greece

Attempt for an alternative synthetic pathway

During the initial investigation of the synthetic procedures, we have also performed the cyclization of **5a** prior the introduction of aniline, in order to have access to the more versatile 6-methylthiopyrazolopyridinol intermediate **12** (Scheme SI1). This compound was converted in the 4-benzyloxyderivative **13** however, our attempts to substitute the 6-methylthiogroup of **13**, or even the corresponding oxidized 6-methylsulfonylderivative **14**, were not successful.

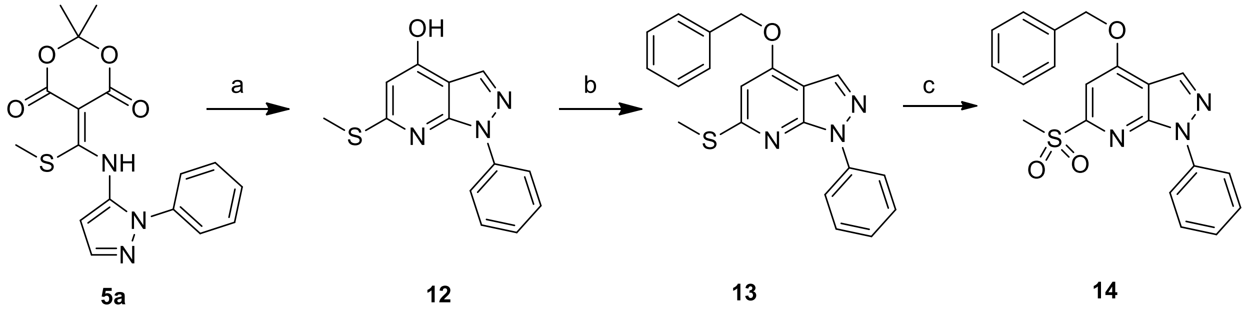

Scheme S1. Reagents and conditions. a) Ph_2O, reflux, Ar, 85%; b) 1)K_2CO_3, DMF, 2) benzylbromide, 50°C, 57%;, c) mCPBA, CH_2Cl_2, rt, 98%.

Experimental data of compounds 12-14

6-(Methylthio)-1-phenyl-*1H*-pyrazolo[3,4-*b*]pyridin-4-ol **(12)**

This derivative was prepared by a method analogous to that described for the synthesis of **7a**. Chromatographic purification (silica gel 40-60μm) was performed using a mixture of cyclohexane / EtOAc (9/1 to 6/4, v/v) as the eluent. Yield 85%. Pale white solid, mp. 154-156°C (EtOAc/*n*-pentane). ^{1}H-NMR (600 MHz, DMSO-d_6) δ (ppm) 2.59 (s, 3H, SCH_3), 6.51 (s, 1H, H-5), 7.31 (t, 1H, *J*= 7.0 Hz, phenyl H-4), 7.54 (t, 2H, *J*= 8.1 Hz, phenyl H-3, H-5), 8.28-8.29 (m, 3H, H-3, phenyl H-2, H-6), 11.78 (brs, 1H, D_2O exch., OH). ^{13}C NMR (151 MHz, DMSO-d_6) δ (ppm) 12.94, 100.38, 106.41, 120.08, 125.58, 129.06, 132.60, 139.37, 151.83, 158.73, 161.77.HR-MS (ESI) m/z calculated for $C_{13}H_{12}N_3OS$ [M+H]$^+$: 258.0696; found258.0696.

6-(Methylthio)-4-benzyloxy-1-phenyl-*1H*-pyrazolo[3,4-*b*]pyridine **(13)**

This derivative was prepared by a method analogous to that described for the synthesis of **8a**. Chromatographic purification (silica gel 40-60μm) was performed using a mixture of cyclohexane / EtOAc (1/1, v/v) as the eluent. Yield 57%. White solid, mp. 138-140°C (CH_2Cl_2/Et_2O). ^{1}H-NMR (600 MHz, $CDCl_3$) δ (ppm) 2.67 (s, 3H, SCH_3), 5.22 (s, 2H, CH_2), 6.54 (s, 1H, H-5), 7.29 (t, 1H, *J*= 7.4 Hz, phenyl H-4), 7.39-7.53 (m, 7H, benzyloxy 5H, phenyl H-3, H-5), 8.12 (s, 1H, H-3), 8.33 (d, 2H, *J*= 7.7 Hz, phenyl H-2,H-6). ^{13}C NMR (151 MHz, $CDCl_3$) δ (ppm) 13.70, 70.69, 98.17, 106.97, 121.01, 125.85, 127.77, 128.70, 128.93, 128.99, 132.14, 135.43, 139.88, 152.09, 159.24, 162.88.HR-MS (ESI) m/z calculated for $C_{20}H_{18}N_3OS$ [M+H]$^+$: 348.1165; found 348.1164.

6-(Methylsulfonyl)-4-benzyloxy-1-phenyl-*1H*-pyrazolo[3,4-*b*]pyridine **(14)**

To a solution of **13** (40 mg, 0.12 mmol) in dichloromethane (4 mL) was added 3-chloroperoxybenzoic acid (m-CPBA, 59 mg, 0.34 mmoL) and the mixture was stirred for 3h at room temperature. It was then neutralized with a Na_2CO_3 solution (150 mg in 40 mL H_2O) and extracted with dichloromethane. The organic layer was washed with brine, dried (Na_2SO_4) and the solvent was vacuum evaporated to provide pure **14** (42 mg, 98%) as a white solid. Mp. 144°C (Et_2O). ^{1}H-NMR (400 MHz, $CDCl_3$) δ (ppm) 3.34 (s, 3H, SO_2CH_3), 5.41 (s, 2H, CH_2), 7.36 (t, 1H, *J*= 7.4 Hz, phenyl H-4), 7.40-7.43 (m, 1H, benzyloxy 1H), 7.46 (t, 2H, *J*= 6.8 Hz, phenyl H-3, H-5), 7.50-7.55 (m, 5H, benzyloxy 4H, H-5), 8.22 (d, 2H, *J*= 7.8 Hz, phenyl H-2, H-6), 8.34 (s, 1H, H-3). ^{13}C NMR (101 MHz, $CDCl_3$) δ (ppm) 39.97, 71.86, 96.50, 110.97, 121.39, 126.85, 128.10, 129.10, 129.15, 129.32, 132.33, 134.48, 139.09, 150.67, 159.32, 161.70.HR-MS (ESI) m/z calculated for $C_{20}H_{18}N_3O_3S$ [M+H]$^+$: 380.1063; found 380.1060.

Spectral data of the new derivatives

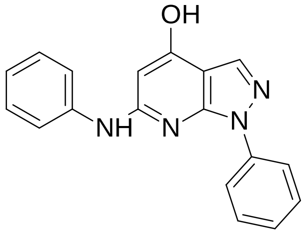

7a

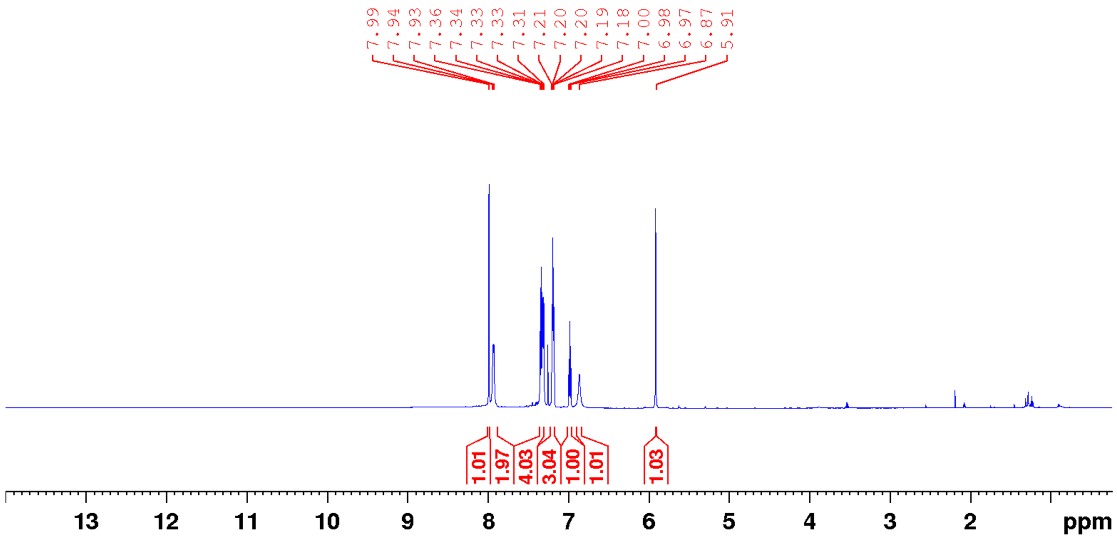

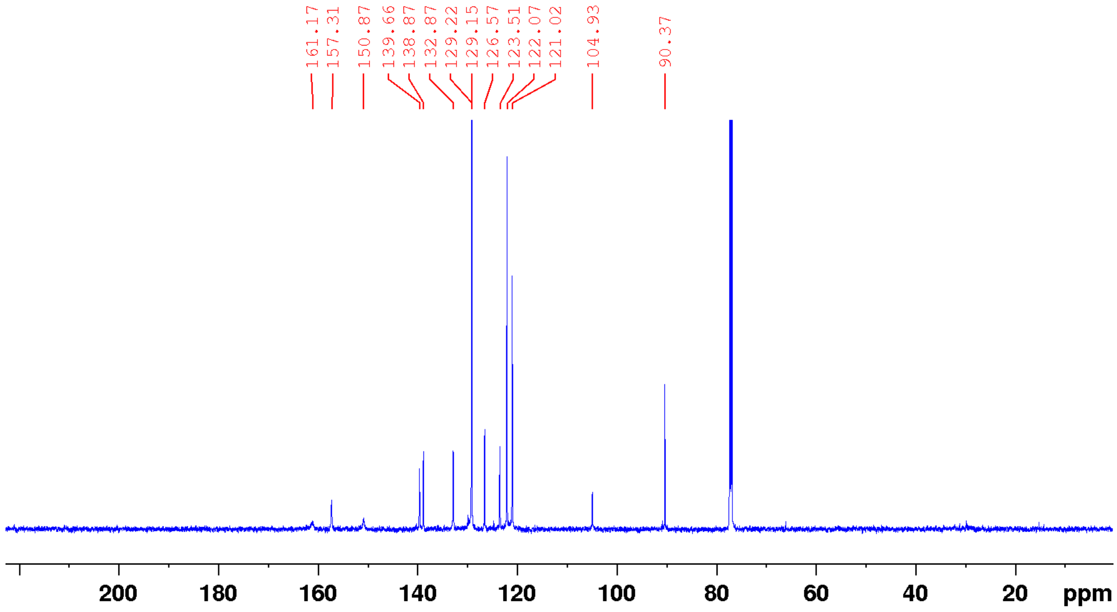

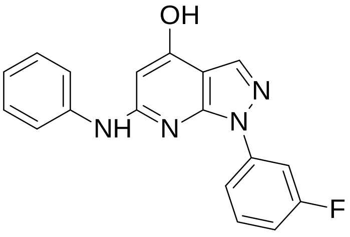
OH
N
N
NH
N
F

7b

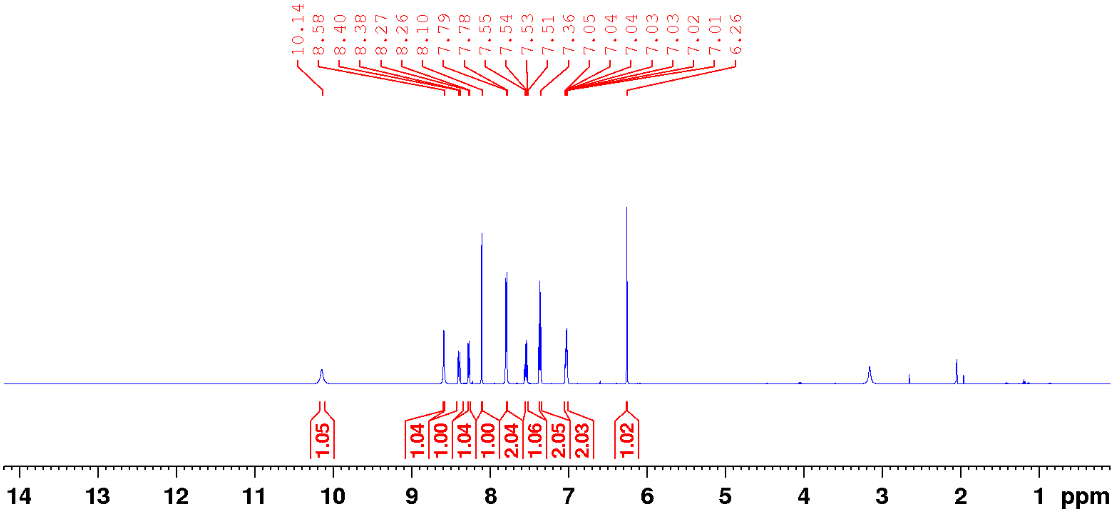
10.14
8.58
8.40
8.38
8.27
8.26
8.10
7.79
7.78
7.55
7.54
7.53
7.51
7.36
7.05
7.04
7.04
7.03
7.03
7.02
7.01
6.26
1.05
1.04
1.00
1.04
1.00
2.04
1.06
2.05
2.03
1.02
14
13
12
11
10
9
8
7
6
5
4
3
2
1
ppm

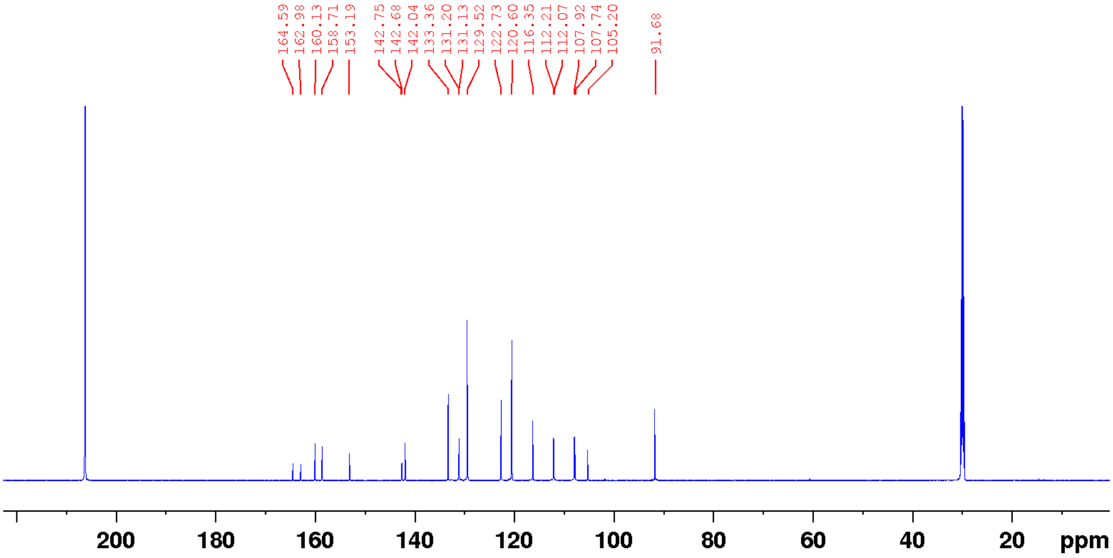
164.59
162.98
160.13
158.71
153.19
142.75
142.68
142.04
133.36
131.20
131.13
129.52
122.73
120.60
116.35
112.21
112.07
107.92
107.74
105.20
91.68
200
180
160
140
120
100
80
60
40
20
ppm

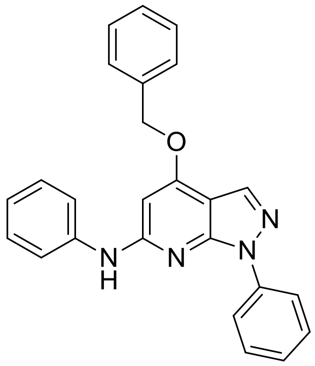

8a

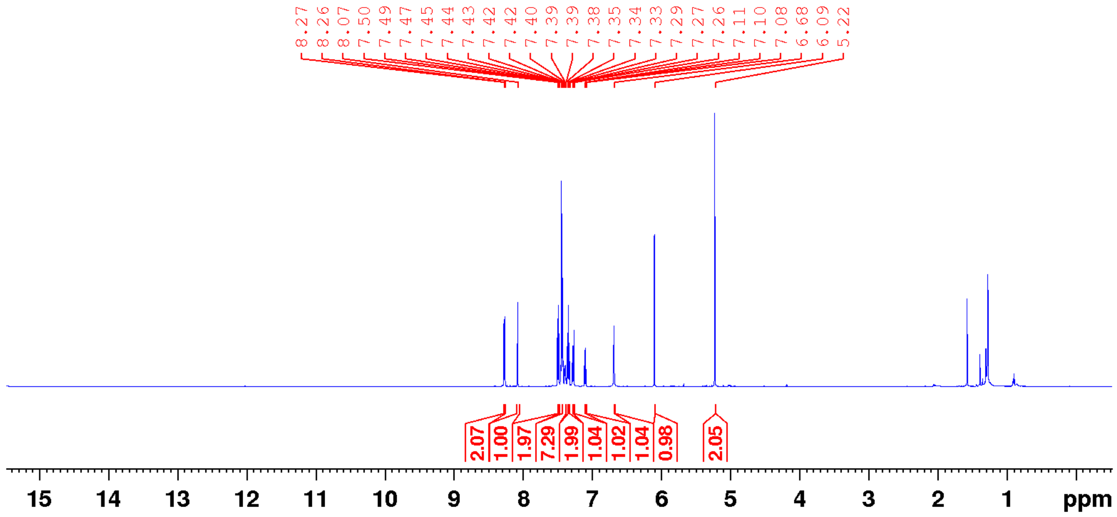
8.27
8.26
8.07
7.50
7.49
7.47
7.45
7.44
7.43
7.42
7.42
7.40
7.39
7.39
7.38
7.35
7.34
7.33
7.29
7.27
7.26
7.11
7.10
7.08
6.68
6.09
5.22
2.07
1.00
1.97
7.29
1.99
1.04
1.02
1.04
0.98
2.05
15
14
13
12
11
10
9
8
7
6
5
4
3
2
1
ppm

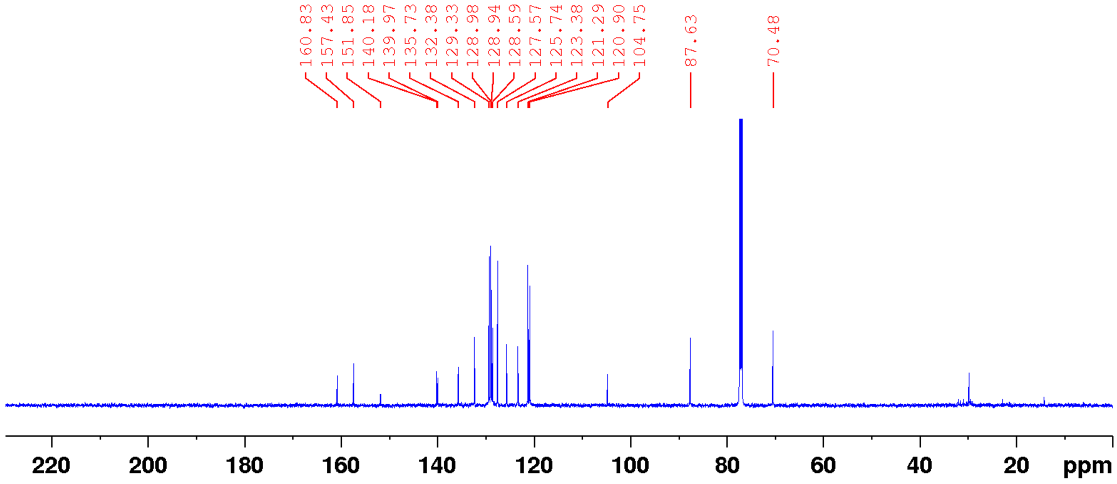
160.83
157.43
151.85
140.18
139.97
135.73
132.38
129.33
128.98
128.94
128.59
127.57
125.74
123.38
121.29
120.90
104.75
87.63
70.48
220
200
180
160
140
120
100
80
60
40
20
ppm

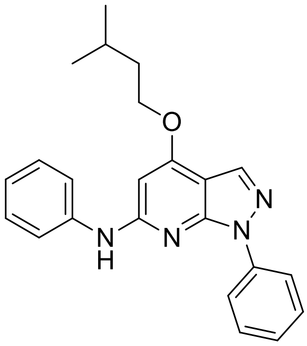

8b

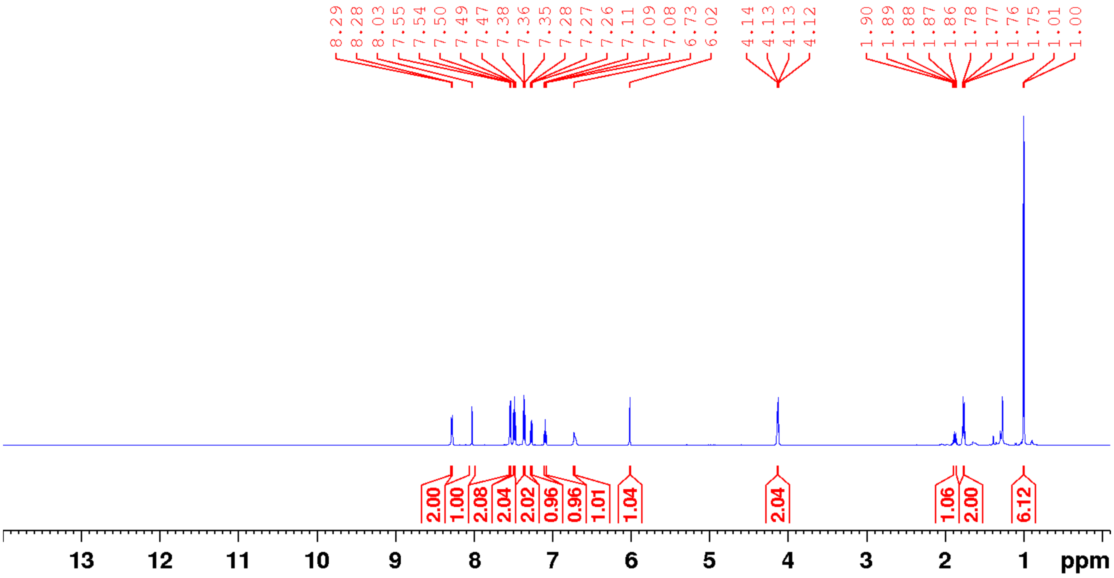

8.29
8.28
8.03
7.55
7.54
7.50
7.49
7.47
7.38
7.36
7.35
7.28
7.27
7.26
7.11
7.09
7.08
6.73
6.02
4.14
4.13
4.13
4.12
1.90
1.89
1.88
1.87
1.86
1.78
1.77
1.76
1.75
1.01
1.00
2.00
1.00
2.08
2.04
2.02
0.96
0.96
1.01
1.04
2.04
1.06
2.00
6.12
13 12 11 10 9 8 7 6 5 4 3 2 1 ppm

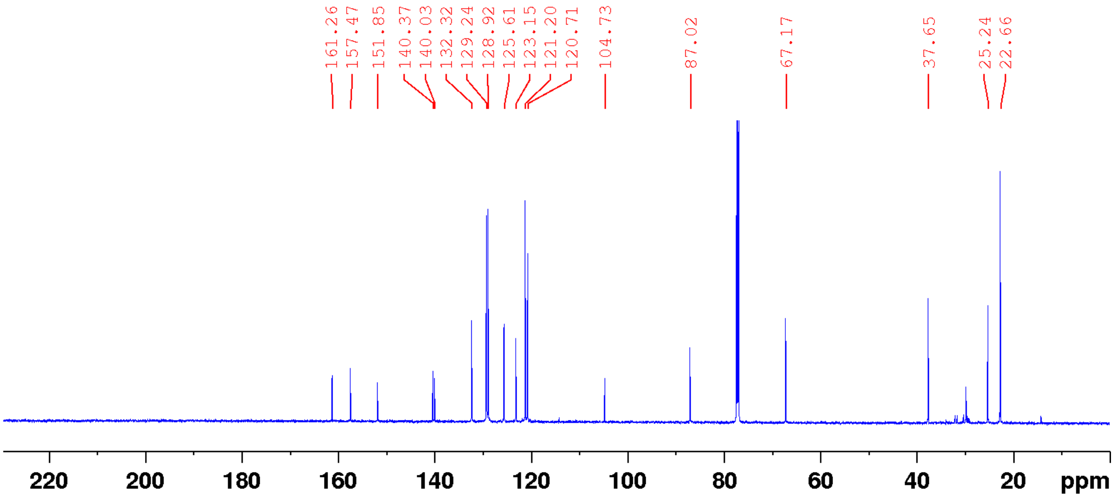

161.26
157.47
151.85
140.37
140.03
132.32
129.24
128.92
125.61
123.15
121.20
120.71
104.73
87.02
67.17
37.65
25.24
22.66
220 200 180 160 140 120 100 80 60 40 20 ppm

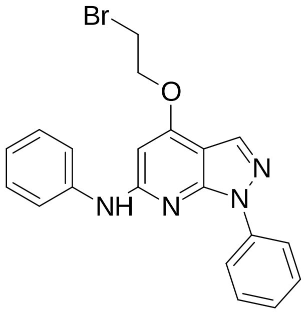
Br
O
N
N
NH
N

8c

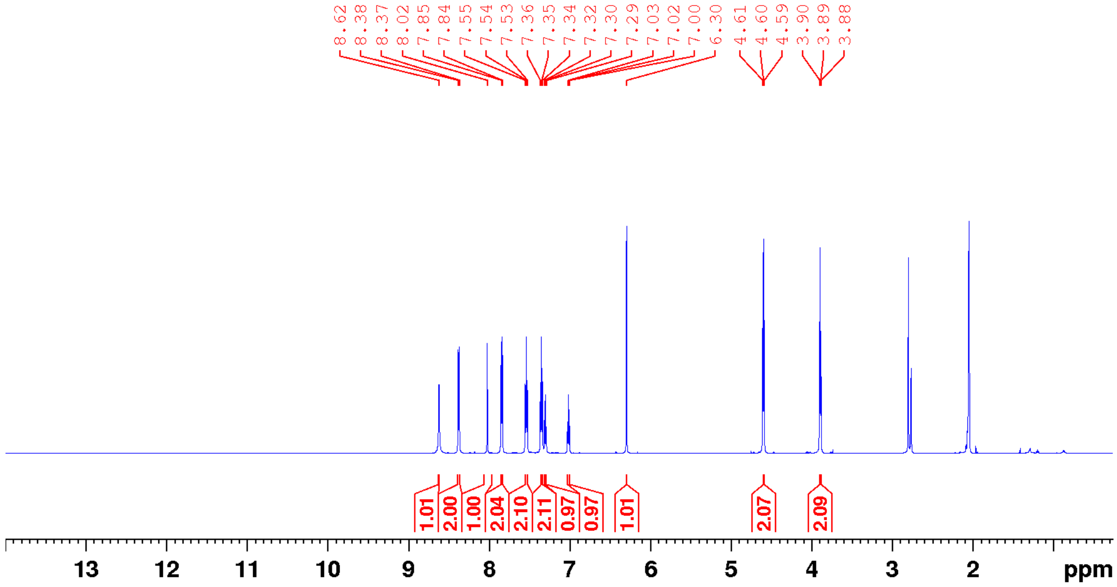
8.62
8.38
8.37
8.02
7.85
7.84
7.55
7.54
7.53
7.36
7.35
7.34
7.32
7.30
7.29
7.03
7.02
7.00
6.30
4.61
4.60
4.59
3.90
3.89
3.88
1.01
2.00
1.00
2.04
2.10
2.11
0.97
0.97
1.01
2.07
2.09
13
12
11
10
9
8
7
6
5
4
3
2
ppm

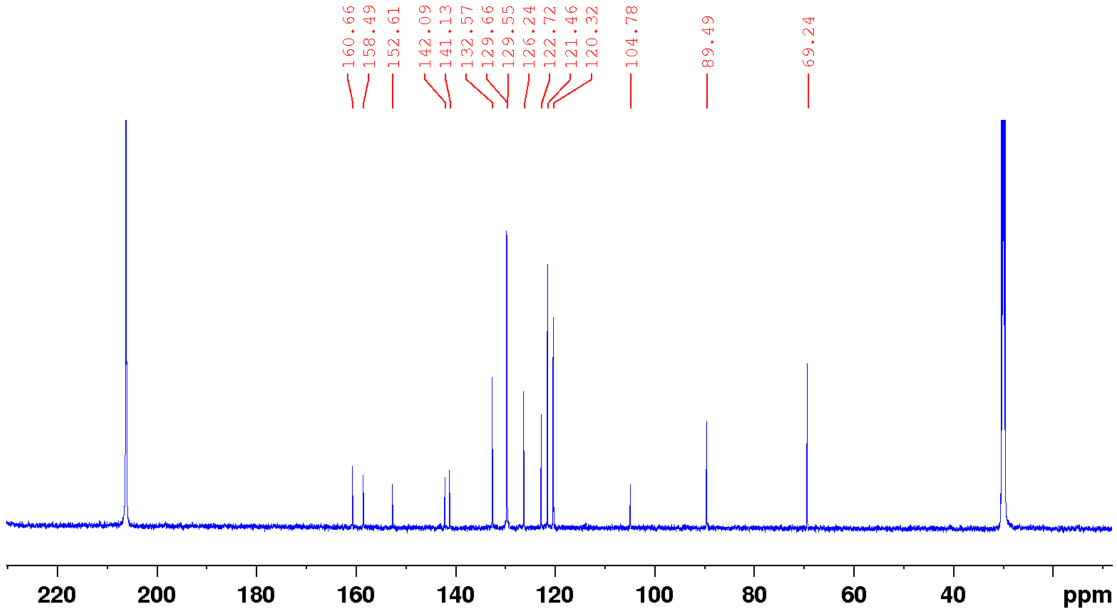
160.66
158.49
152.61
142.09
141.13
132.57
129.66
129.55
126.24
122.72
121.46
120.32
104.78
89.49
69.24
220
200
180
160
140
120
100
80
60
40
ppm

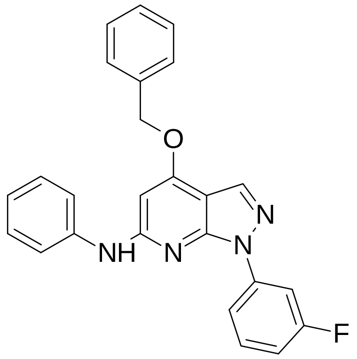
O
N
N
NH
N
F

8d

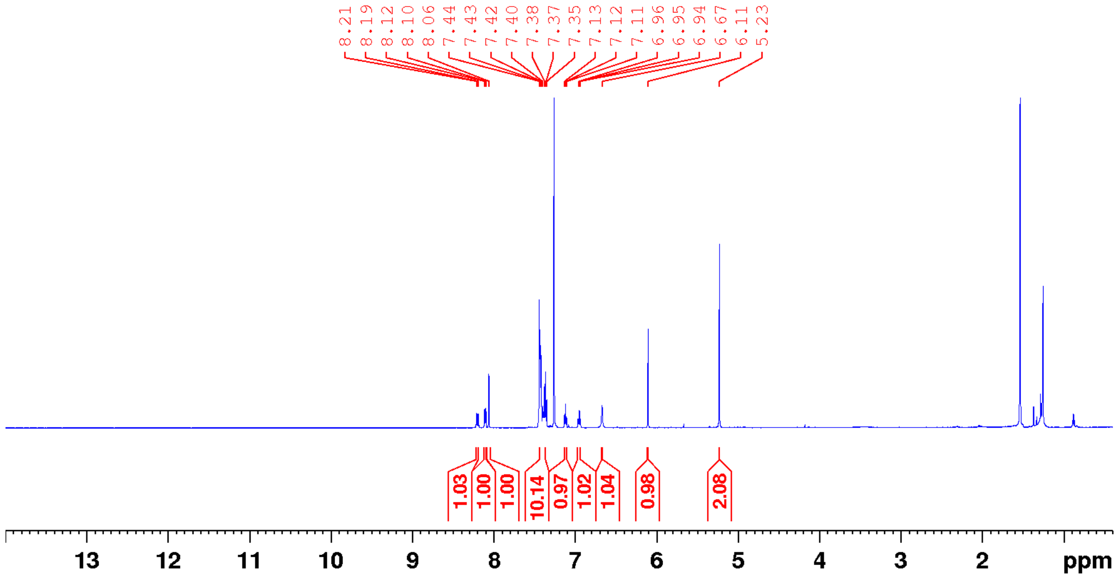
8.21 8.19 8.12 8.10 8.06 7.44 7.43 7.42 7.40 7.38 7.37 7.35 7.13 7.12 7.11 6.96 6.95 6.94 6.67 6.11 5.23
1.03 1.00 1.00 10.14 0.97 1.02 1.04 0.98 2.08
13 12 11 10 9 8 7 6 5 4 3 2 ppm

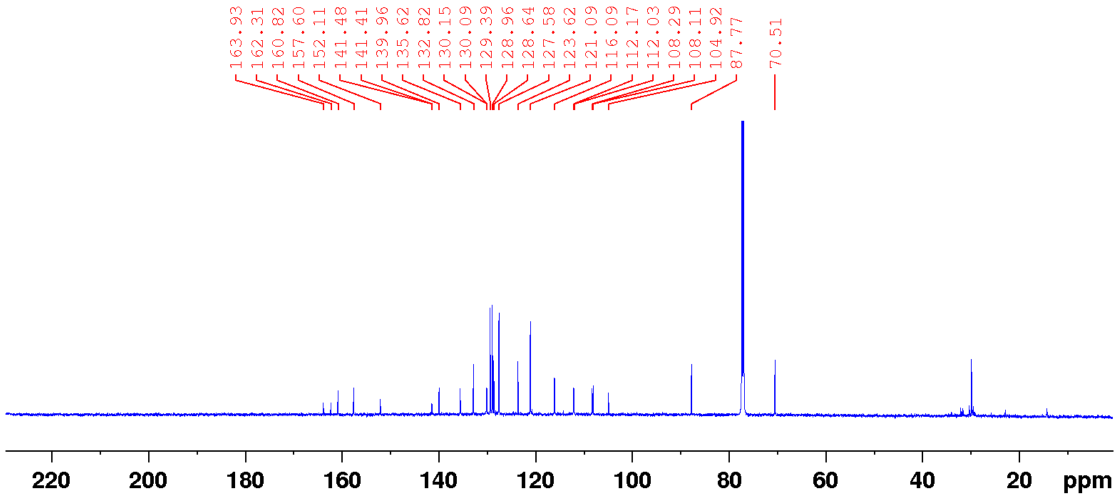
163.93 162.31 160.82 157.60 152.11 141.48 141.41 139.96 135.62 132.82 130.15 130.09 129.39 128.96 128.64 127.58 123.62 121.09 116.09 112.17 112.03 108.29 108.11 104.92 87.77
70.51
220 200 180 160 140 120 100 80 60 40 20 ppm

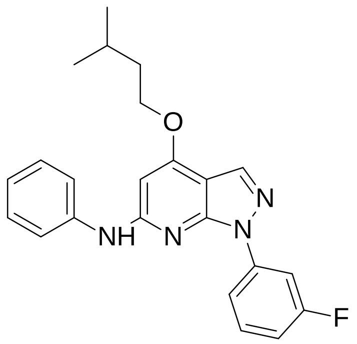
O
N
N
NH
N
F

8e

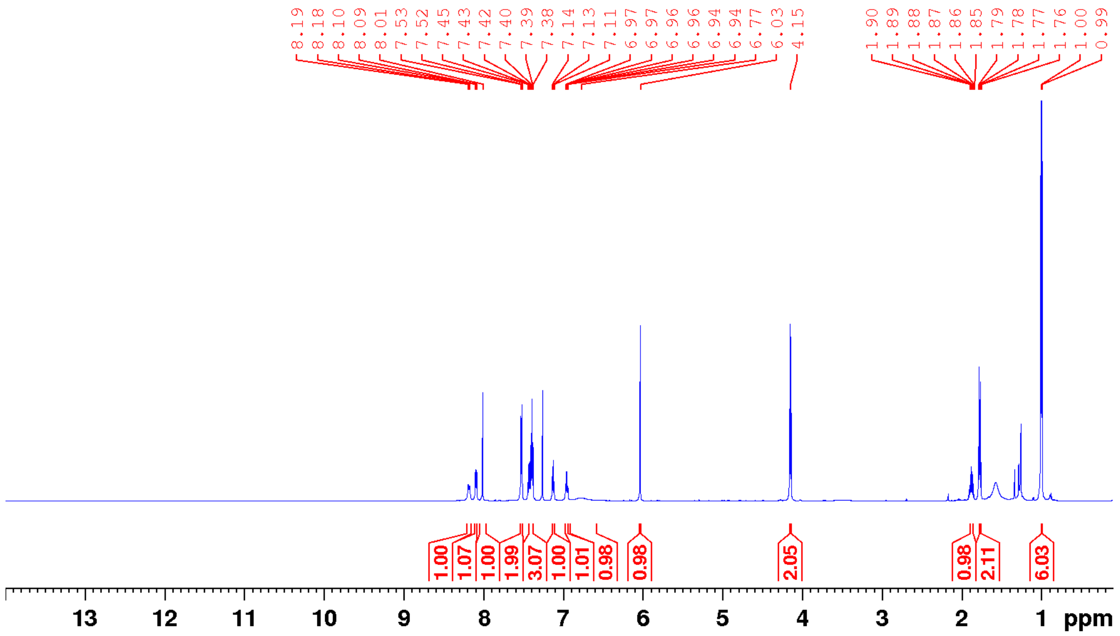
8.19 8.18 8.10 8.09 8.01 7.53 7.52 7.45 7.43 7.42 7.40 7.39 7.38 7.14 7.13 7.11 6.97 6.97 6.96 6.94 6.94 6.77 6.03 4.15
1.90 1.89 1.88 1.87 1.86 1.85 1.79 1.78 1.77 1.76 1.00 0.99
1.00 1.07 1.00 1.99 3.07 1.00 1.01 0.98 0.98 2.05 0.98 2.11 6.03
13 12 11 10 9 8 7 6 5 4 3 2 1 ppm

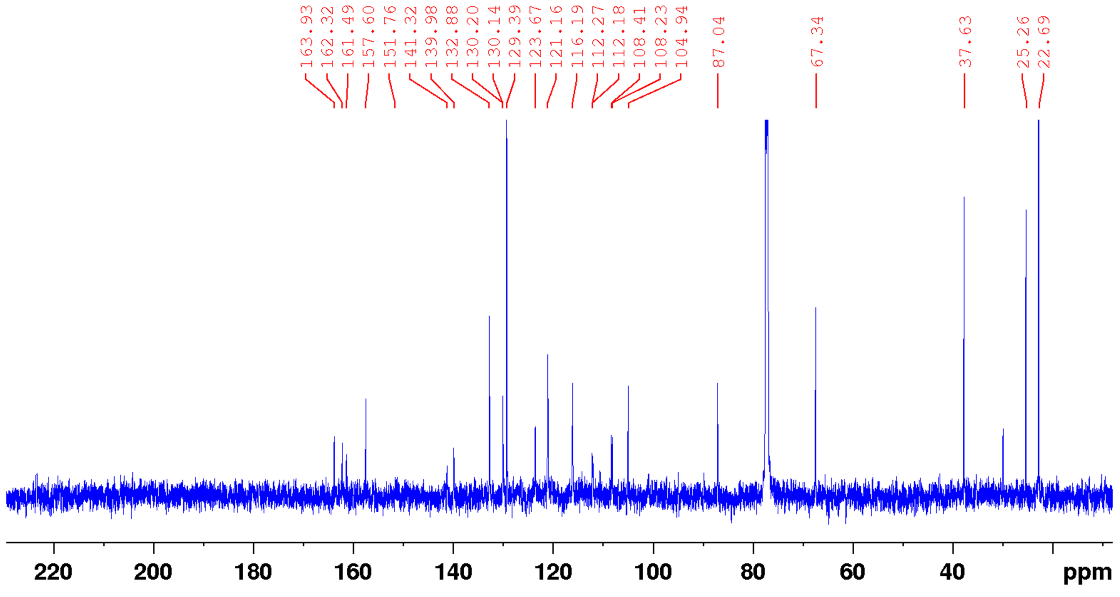
163.93 162.32 161.49 157.60 151.76 141.32 139.98 132.88 130.20 130.14 129.39 123.67 121.16 116.19 112.27 112.18 108.41 108.23 104.94 87.04 67.34 37.63 25.26 22.69
220 200 180 160 140 120 100 80 60 40 ppm

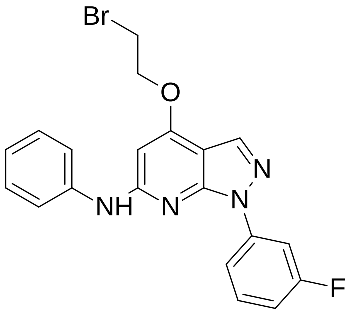

Br
O
N
N
NH
N
F

8f

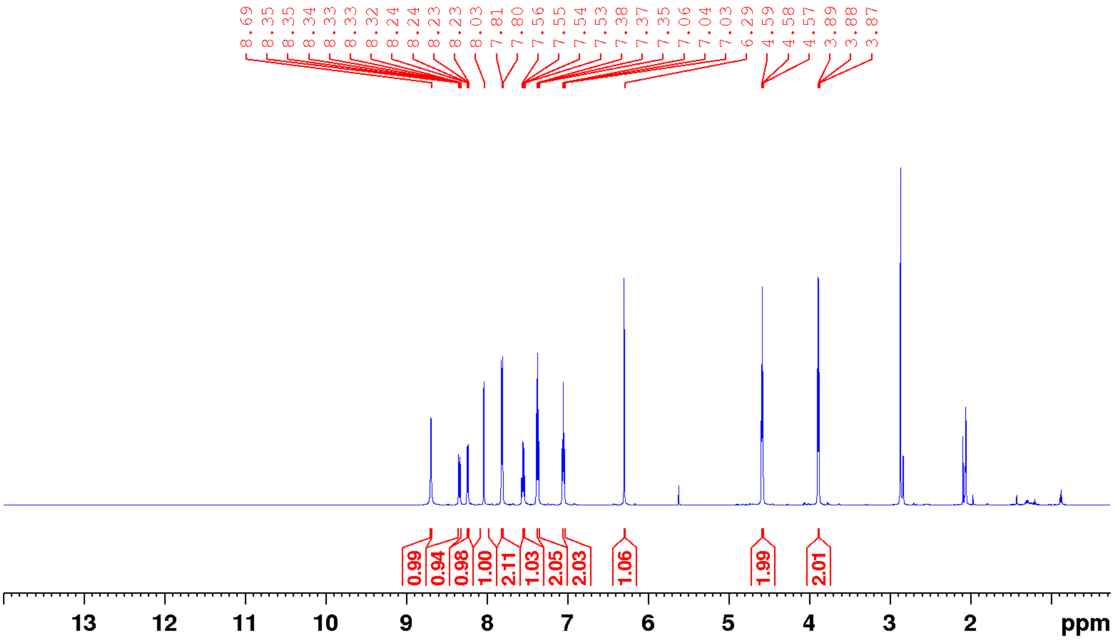

8.69
8.35
8.35
8.34
8.33
8.33
8.33
8.32
8.24
8.24
8.23
8.23
8.03
7.81
7.80
7.56
7.55
7.54
7.53
7.38
7.37
7.35
7.06
7.04
7.03
6.29
4.59
4.58
4.57
3.89
3.88
3.87
0.99
0.94
0.98
1.00
2.11
1.03
2.05
2.03
1.06
1.99
2.01
13
12
11
10
9
8
7
6
5
4
3
2
ppm

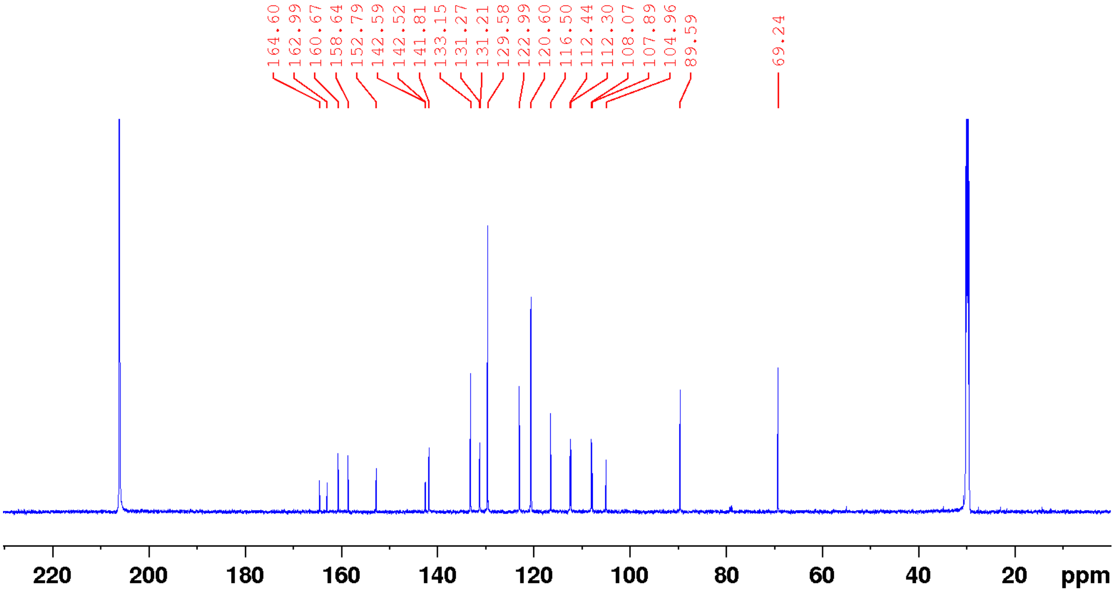

164.60
162.99
160.67
158.64
152.79
142.59
142.52
141.81
133.15
131.27
131.21
129.58
122.99
120.60
116.50
112.44
112.30
108.07
107.89
104.96
89.59
69.24
220
200
180
160
140
120
100
80
60
40
20
ppm

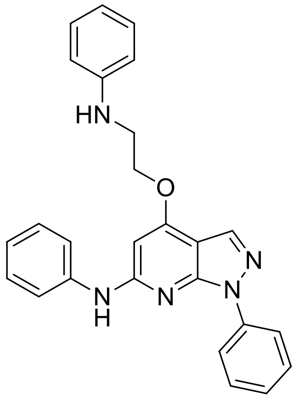
HN
O
N
N
N
N
H

9a

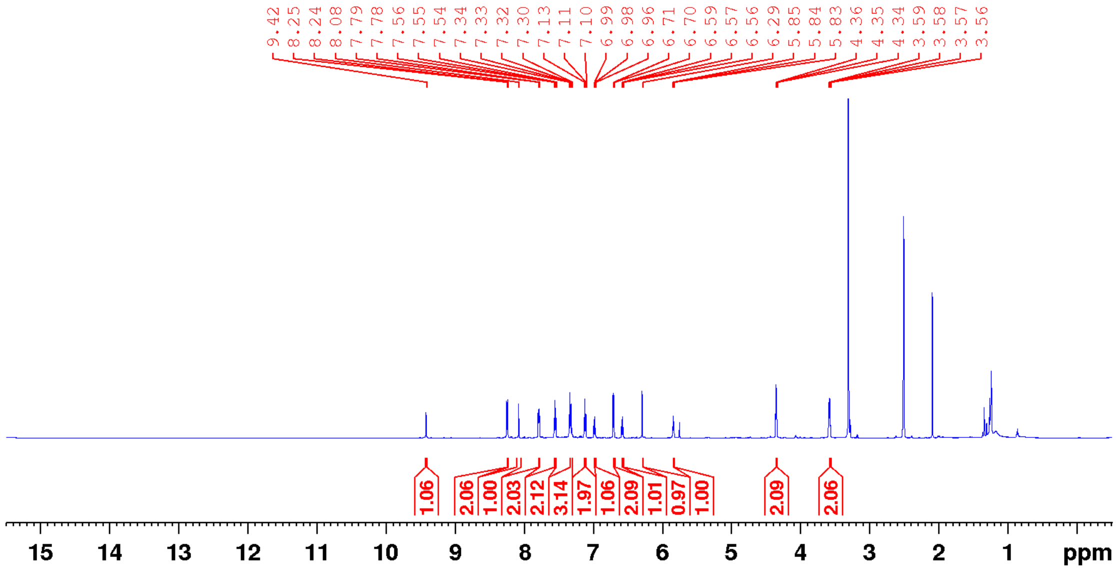
9.42 8.25 8.24 8.08 7.79 7.78 7.56 7.55 7.54 7.34 7.33 7.32 7.30 7.13 7.11 7.10 6.99 6.98 6.96 6.71 6.70 6.59 6.57 6.56 6.29 5.85 5.84 5.83 4.36 4.35 4.34 3.59 3.58 3.57 3.56
1.06 2.06 1.00 2.03 2.12 3.14 1.97 1.06 2.09 1.01 0.97 1.00 2.09 2.06
15 14 13 12 11 10 9 8 7 6 5 4 3 2 1 ppm

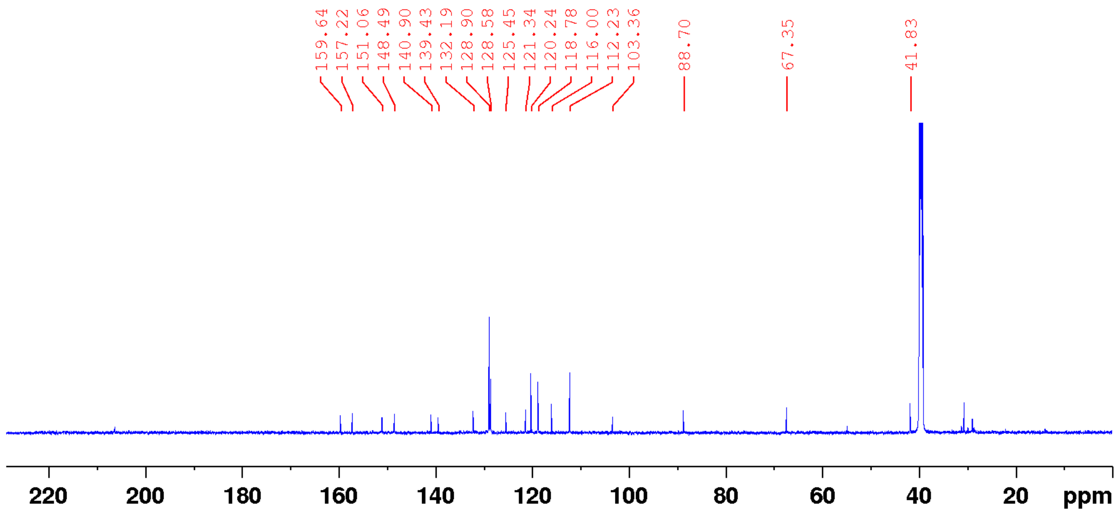
159.64 157.22 151.06 148.49 140.90 139.43 132.19 128.90 128.58 125.45 121.34 120.24 118.78 116.00 112.23 103.36 88.70 67.35 41.83
220 200 180 160 140 120 100 80 60 40 20 ppm

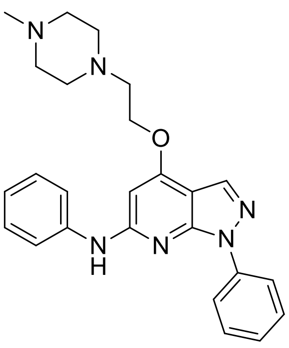
N
N
O
N
N
N
N
H

9b

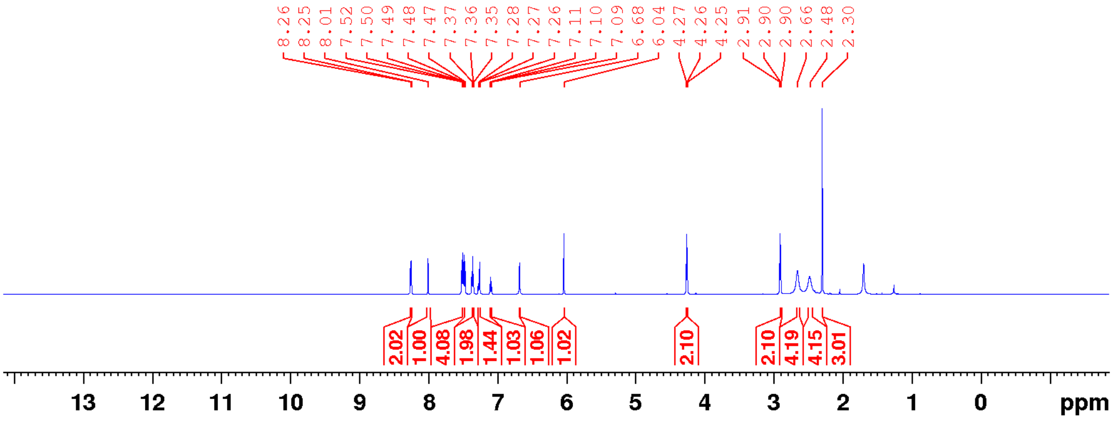
8.26
8.25
8.01
7.52
7.50
7.49
7.48
7.47
7.37
7.36
7.35
7.28
7.27
7.26
7.11
7.10
7.09
6.68
6.04
4.27
4.26
4.25
2.91
2.90
2.90
2.66
2.48
2.30
2.02
1.00
4.08
1.98
1.44
1.03
1.06
1.02
2.10
2.10
4.19
4.15
3.01
13
12
11
10
9
8
7
6
5
4
3
2
1
0
ppm

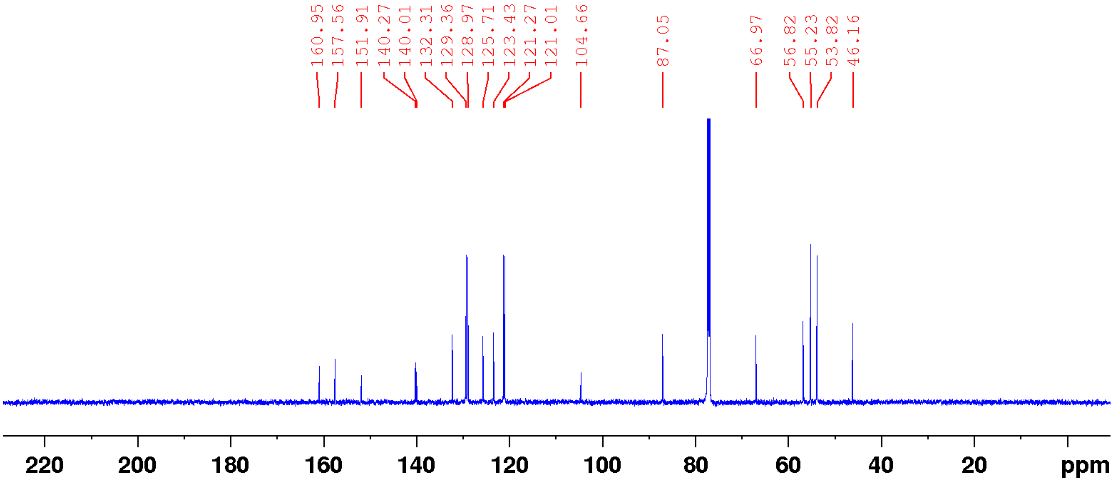
160.95
157.56
151.91
140.27
140.01
132.31
129.36
128.97
125.71
123.43
121.27
121.01
104.66
87.05
66.97
56.82
55.23
53.82
46.16
220
200
180
160
140
120
100
80
60
40
20
ppm

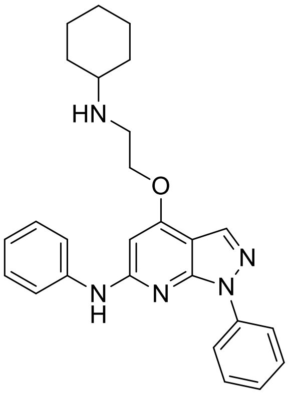
HN
O
N
N
N
N
H

9c

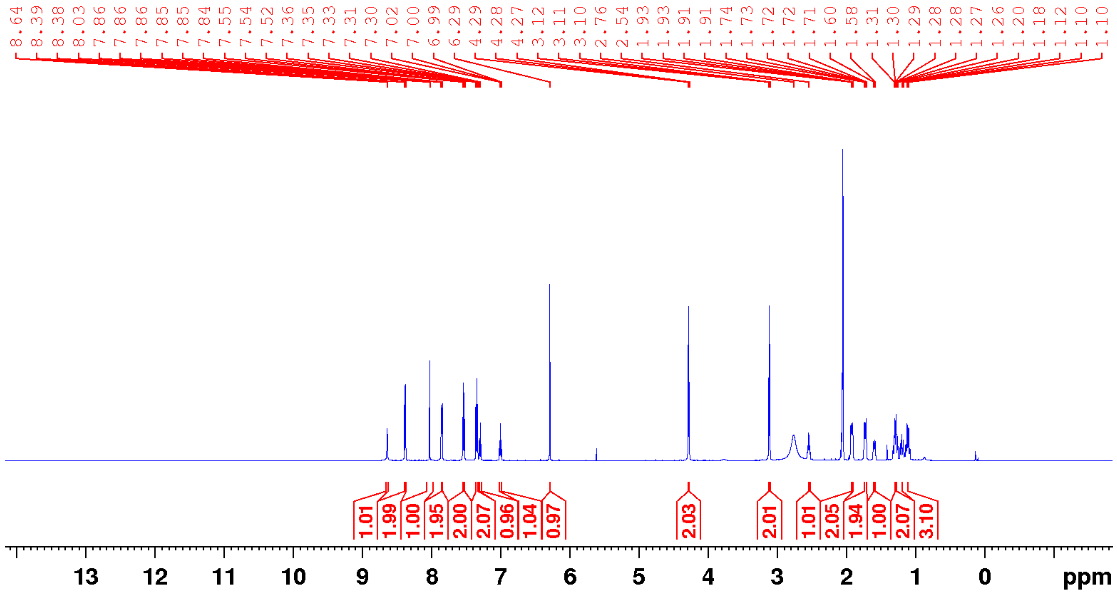
8.64 8.39 8.38 8.03 7.86 7.86 7.86 7.85 7.85 7.84 7.55 7.54 7.52 7.36 7.35 7.33 7.31 7.30 7.02 7.00 6.99 6.29 4.29 4.28 4.27 3.12 3.11 3.10 2.76 2.54 1.93 1.93 1.91 1.91 1.74 1.73 1.72 1.72 1.71 1.60 1.58 1.31 1.30 1.29 1.28 1.28 1.27 1.26 1.20 1.18 1.12 1.10 1.10
1.01 1.99 1.00 1.95 2.00 2.07 0.96 1.04 0.97 2.03 2.01 1.01 2.05 1.94 1.00 2.07 3.10
13 12 11 10 9 8 7 6 5 4 3 2 1 0 ppm

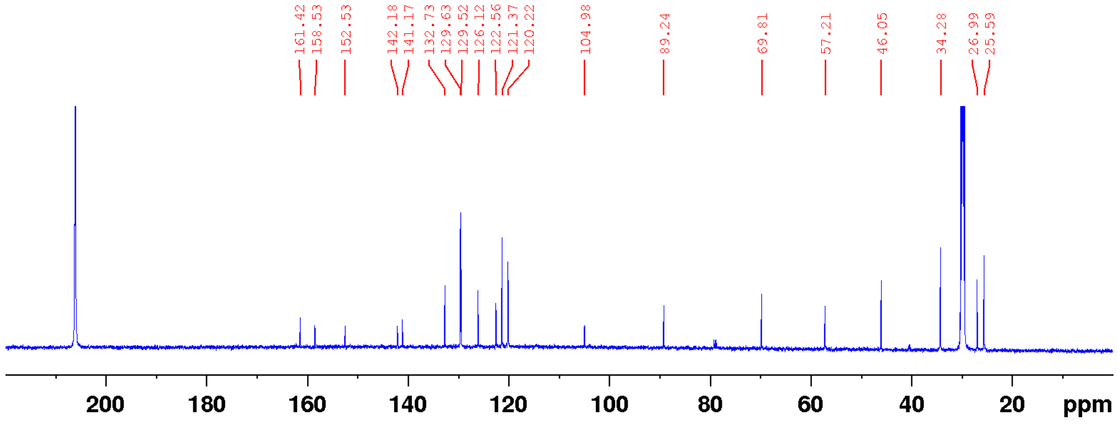
161.42 158.53 152.53 142.18 141.17 132.73 129.63 129.52 126.12 122.56 121.37 120.22 104.98 89.24 69.81 57.21 46.05 34.28 26.99 25.59
200 180 160 140 120 100 80 60 40 20 ppm

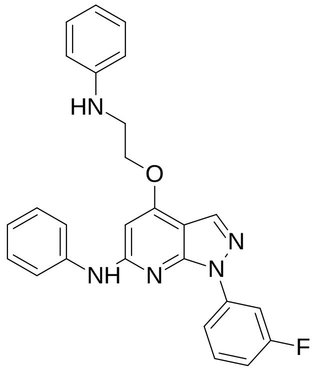

HN
O
N
NH
N
N
F

9d

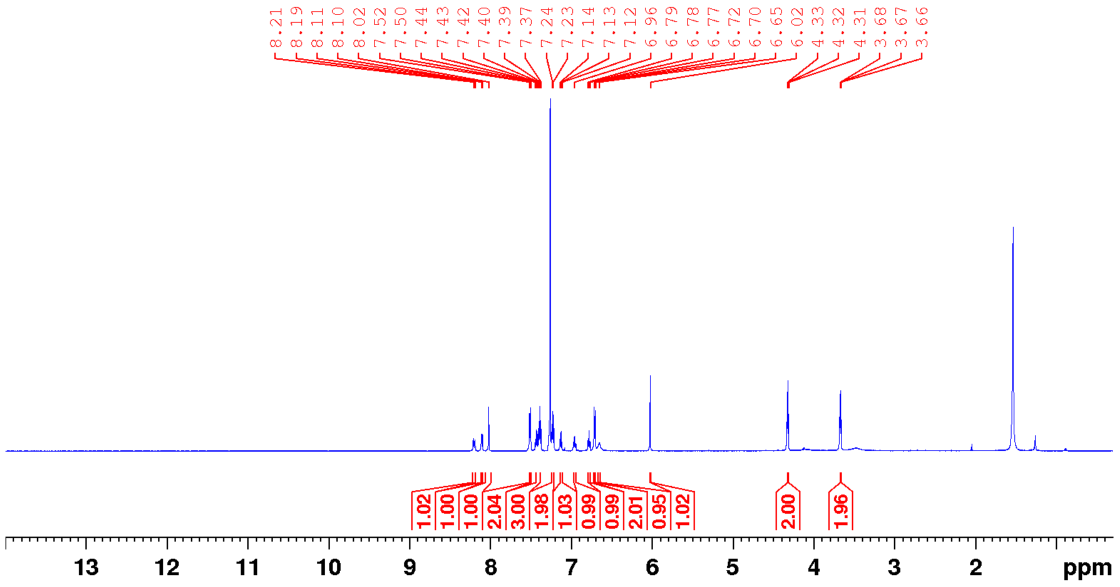

8.21
8.19
8.11
8.10
8.02
7.52
7.50
7.44
7.43
7.42
7.40
7.39
7.37
7.24
7.23
7.14
7.13
7.12
6.96
6.79
6.78
6.77
6.72
6.70
6.65
6.02
4.33
4.32
4.31
3.68
3.67
3.66
1.02
1.00
1.00
2.04
3.00
1.98
1.03
0.99
0.99
2.01
0.95
1.02
2.00
1.96
13
12
11
10
9
8
7
6
5
4
3
2
ppm

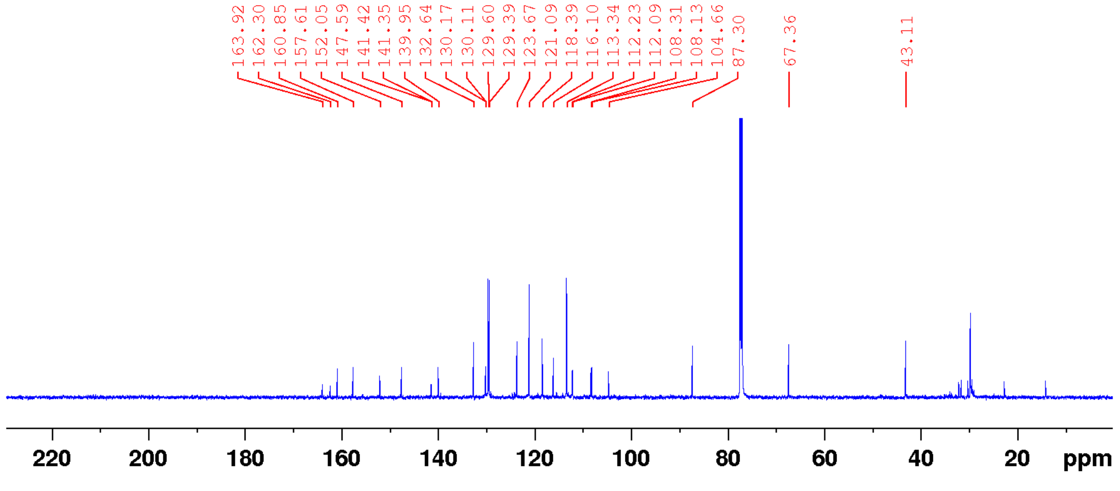

163.92
162.30
160.85
157.61
152.05
147.59
141.42
141.35
139.95
132.64
130.17
130.11
129.60
129.39
123.67
121.09
118.39
116.10
113.34
112.23
112.09
108.31
108.13
104.66
87.30
67.36
43.11
220
200
180
160
140
120
100
80
60
40
20
ppm

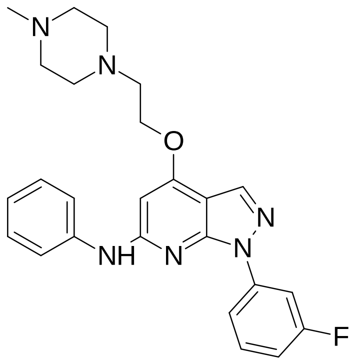
N
N
O
N
N
NH
N
F

9e

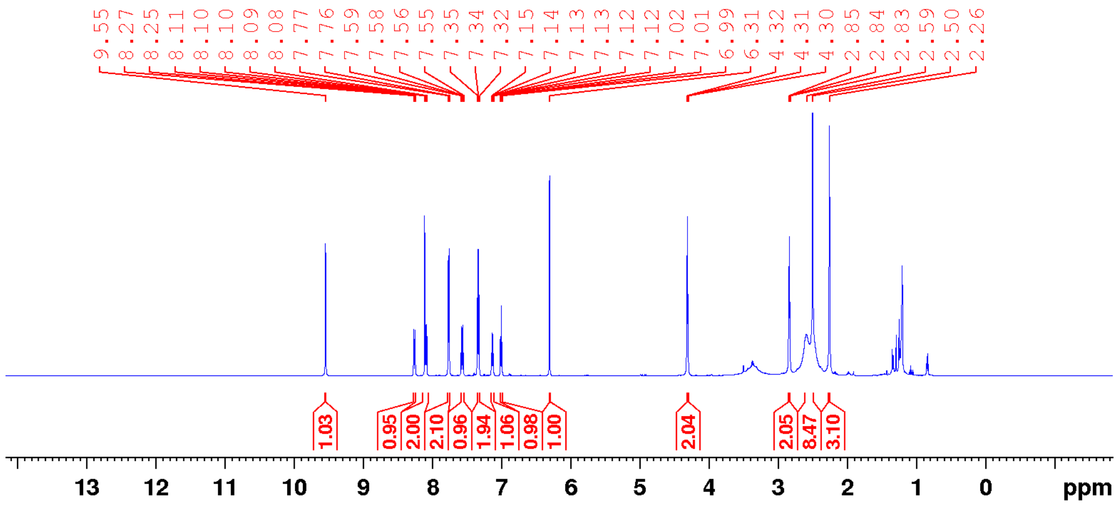
9.55
8.27
8.25
8.11
8.10
8.10
8.09
8.08
7.77
7.76
7.59
7.58
7.56
7.55
7.35
7.34
7.32
7.15
7.14
7.13
7.13
7.12
7.12
7.02
7.01
6.99
6.31
4.32
4.31
4.30
2.85
2.84
2.83
2.59
2.50
2.26
1.03
0.95
2.00
2.10
0.96
1.94
1.06
0.98
1.00
2.04
2.05
8.47
3.10
13
12
11
10
9
8
7
6
5
4
3
2
1
0
ppm

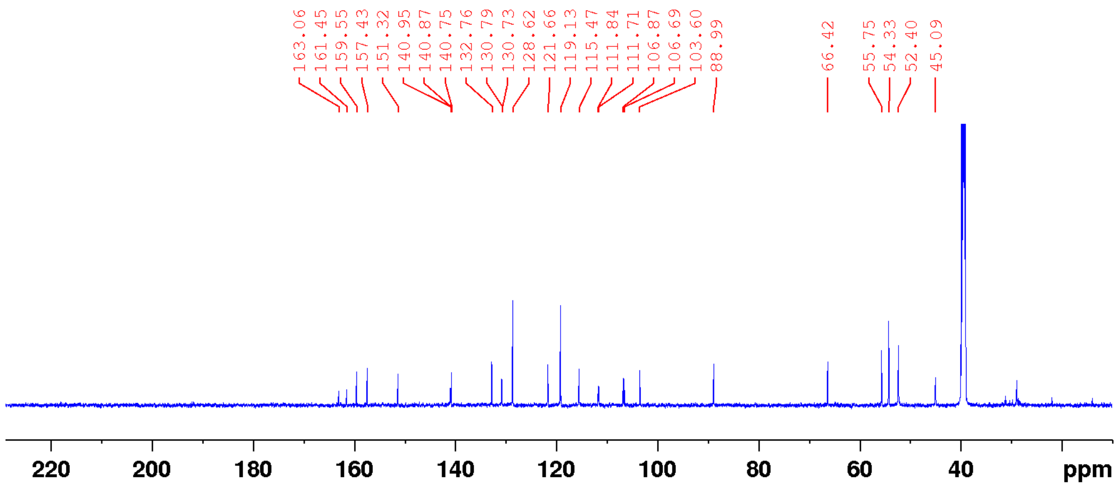
163.06
161.45
159.55
157.43
151.32
140.95
140.87
140.75
132.76
130.79
130.73
128.62
121.66
119.13
115.47
111.84
111.71
106.87
106.69
103.60
88.99
66.42
55.75
54.33
52.40
45.09
220
200
180
160
140
120
100
80
60
40
ppm

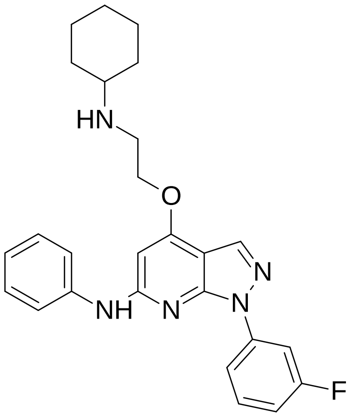
HN
O
N
N
NH
N
F

9f

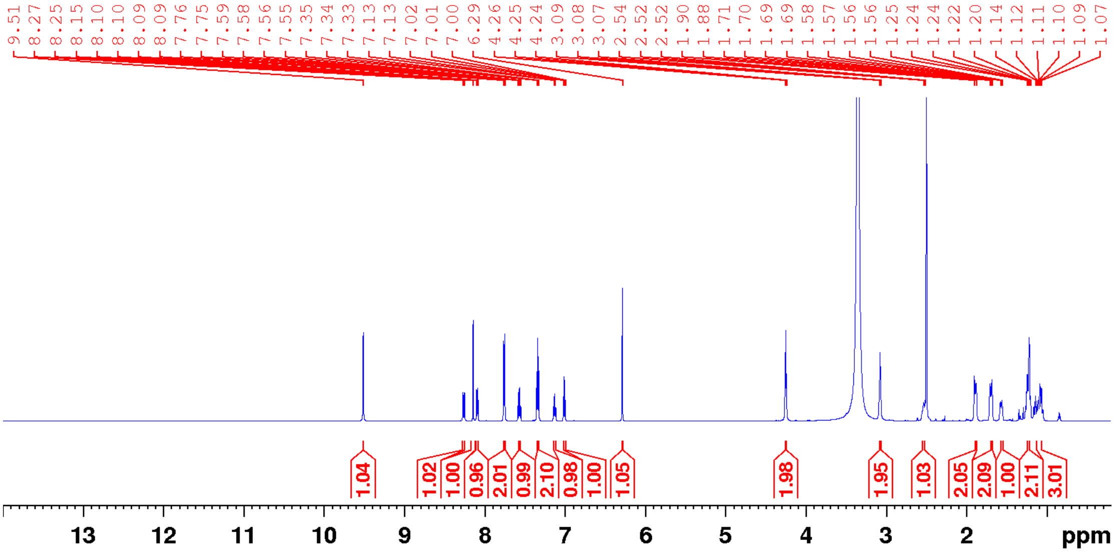
9.51 8.27 8.25 8.15 8.10 8.10 8.09 8.09 7.76 7.75 7.59 7.58 7.56 7.55 7.35 7.34 7.33 7.13 7.13 7.02 7.01 7.00 6.29 4.26 4.25 4.24 3.09 3.08 3.07 2.54 2.52 2.52 1.90 1.88 1.71 1.70 1.69 1.69 1.58 1.57 1.56 1.56 1.25 1.24 1.24 1.22 1.20 1.14 1.12 1.11 1.10 1.09 1.07
1.04 1.02 1.00 0.96 2.01 0.99 2.10 0.98 1.00 1.05 1.98 1.95 1.03 2.05 2.09 1.00 2.11 3.01
13 12 11 10 9 8 7 6 5 4 3 2 ppm

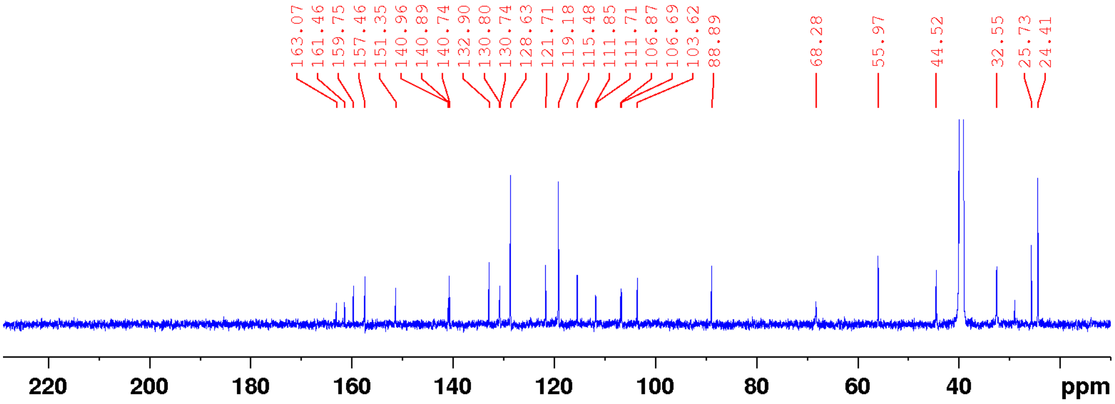
163.07 161.46 159.75 157.46 151.35 140.96 140.89 140.74 132.90 130.80 130.74 128.63 121.71 119.18 115.48 111.85 111.71 106.87 106.69 103.62 88.89 68.28 55.97 44.52 32.55 25.73 24.41
220 200 180 160 140 120 100 80 60 40 ppm

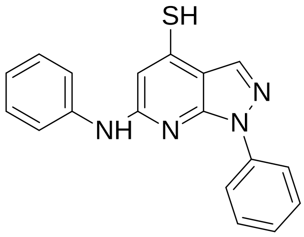
SH
N
N
NH
N

10a

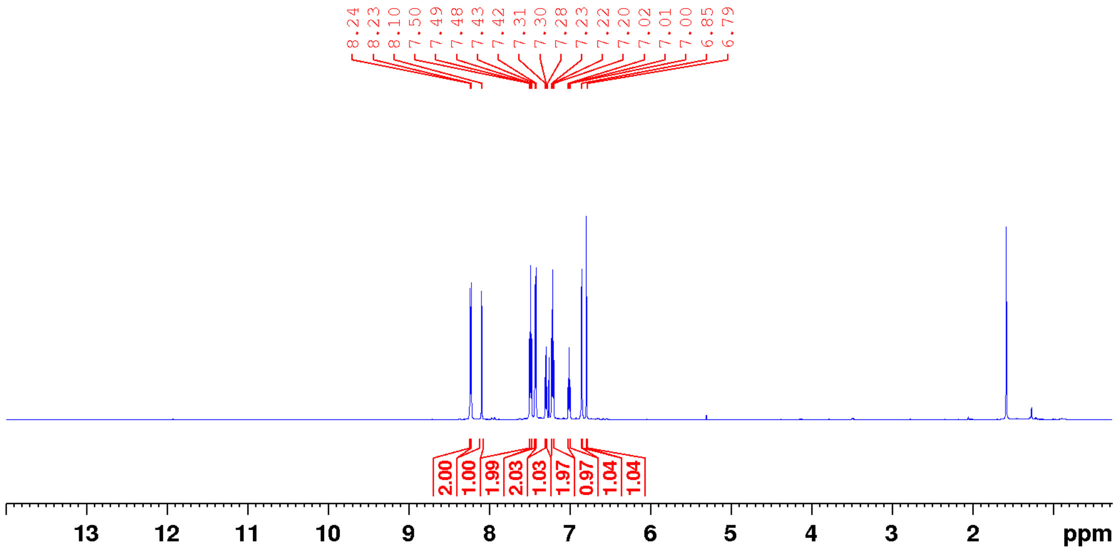
8.24
8.23
8.10
7.50
7.49
7.48
7.43
7.42
7.31
7.30
7.28
7.23
7.22
7.20
7.02
7.01
7.00
6.85
6.79
2.00
1.00
1.99
2.03
1.03
1.97
0.97
1.04
1.04
13
12
11
10
9
8
7
6
5
4
3
2
ppm

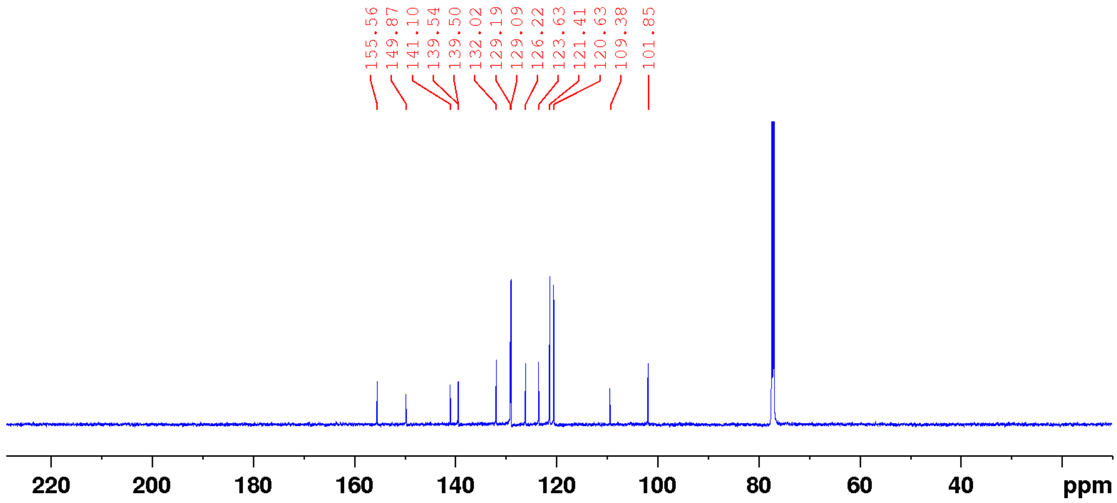
155.56
149.87
141.10
139.54
139.50
132.02
129.19
129.09
126.22
123.63
121.41
120.63
109.38
101.85
220
200
180
160
140
120
100
80
60
40
ppm

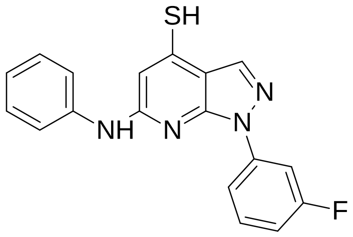
SH
N
NH
N
N
F

10b

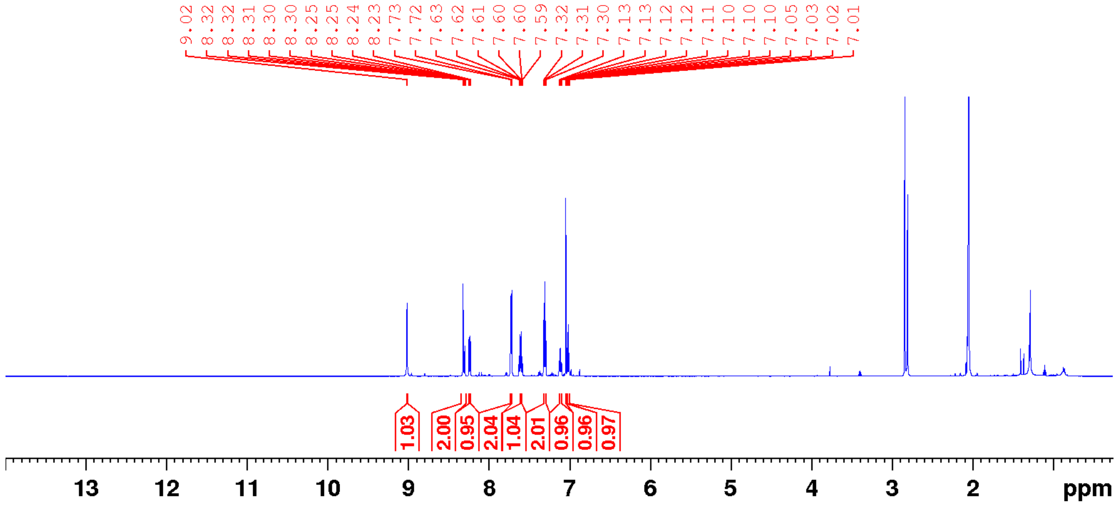
9.02 8.32 8.32 8.31 8.30 8.30 8.25 8.25 8.24 8.23 7.73 7.72 7.63 7.62 7.61 7.60 7.60 7.59 7.32 7.31 7.30 7.13 7.13 7.12 7.12 7.11 7.10 7.10 7.10 7.05 7.03 7.02 7.01
1.03 2.00 0.95 2.04 1.04 2.01 0.96 0.96 0.97
13 12 11 10 9 8 7 6 5 4 3 2 ppm

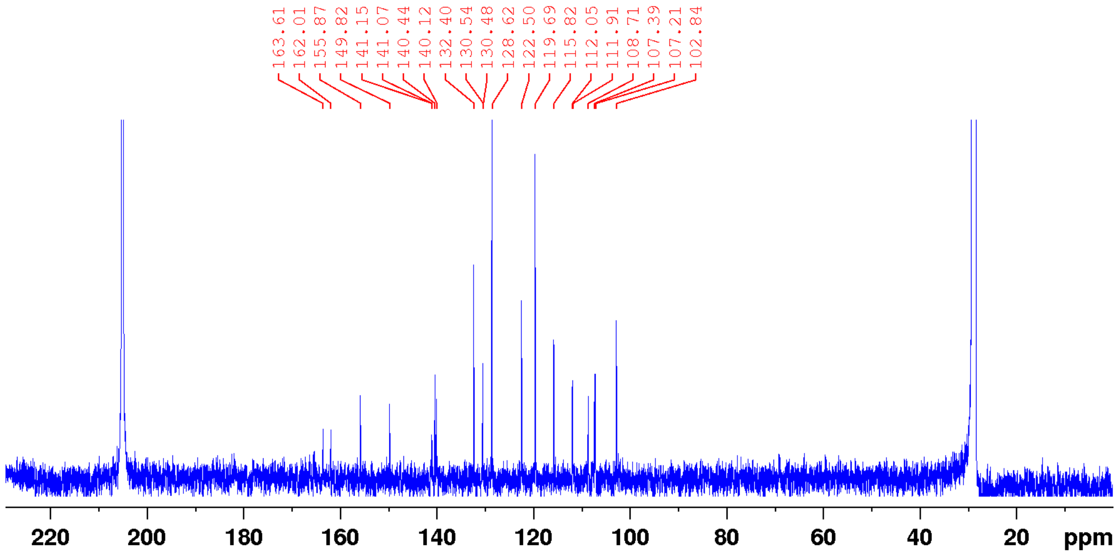
163.61 162.01 155.87 149.82 141.15 141.07 140.44 140.12 132.40 130.54 130.48 128.62 122.50 119.69 115.82 112.05 111.91 108.71 107.39 107.21 102.84
220 200 180 160 140 120 100 80 60 40 20 ppm

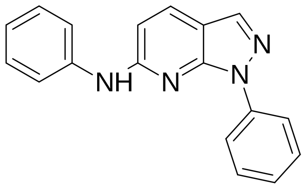

11a

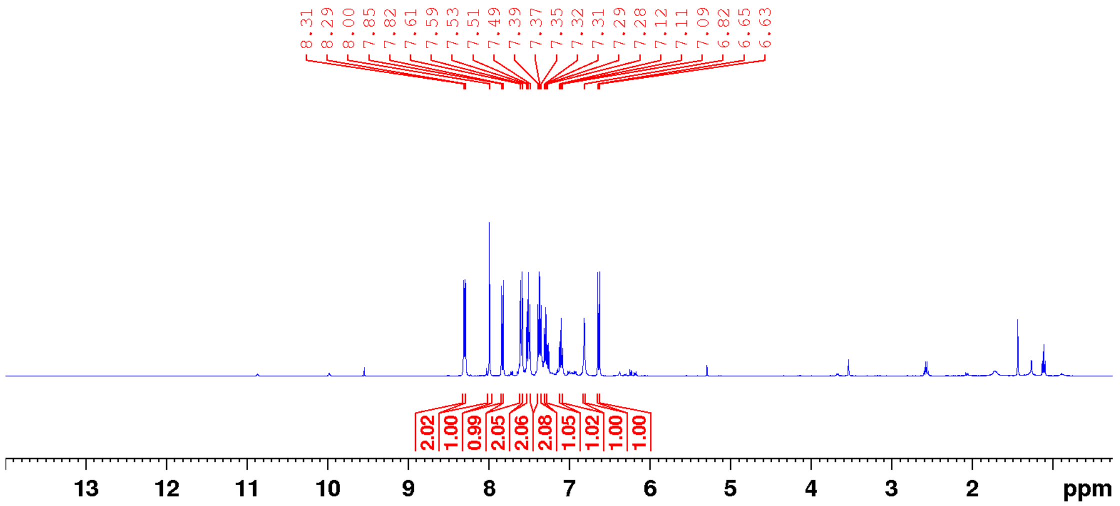

8.31
8.29
8.00
7.85
7.82
7.61
7.59
7.53
7.51
7.49
7.39
7.37
7.35
7.32
7.31
7.29
7.28
7.12
7.11
7.09
6.82
6.65
6.63
2.02
1.00
0.99
2.05
2.06
2.08
1.05
1.02
1.00
1.00
13
12
11
10
9
8
7
6
5
4
3
2
ppm

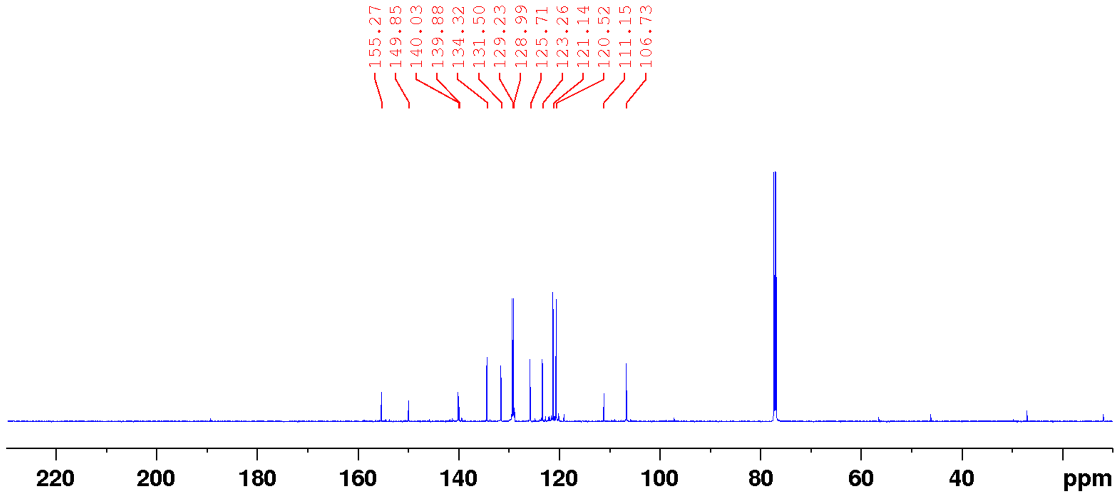

155.27
149.85
140.03
139.88
134.32
131.50
129.23
128.99
125.71
123.26
121.14
120.52
111.15
106.73
220
200
180
160
140
120
100
80
60
40
ppm

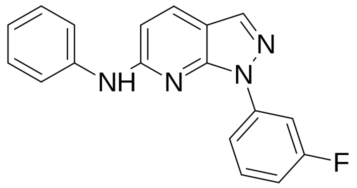
NH
N
N
N
F

11b

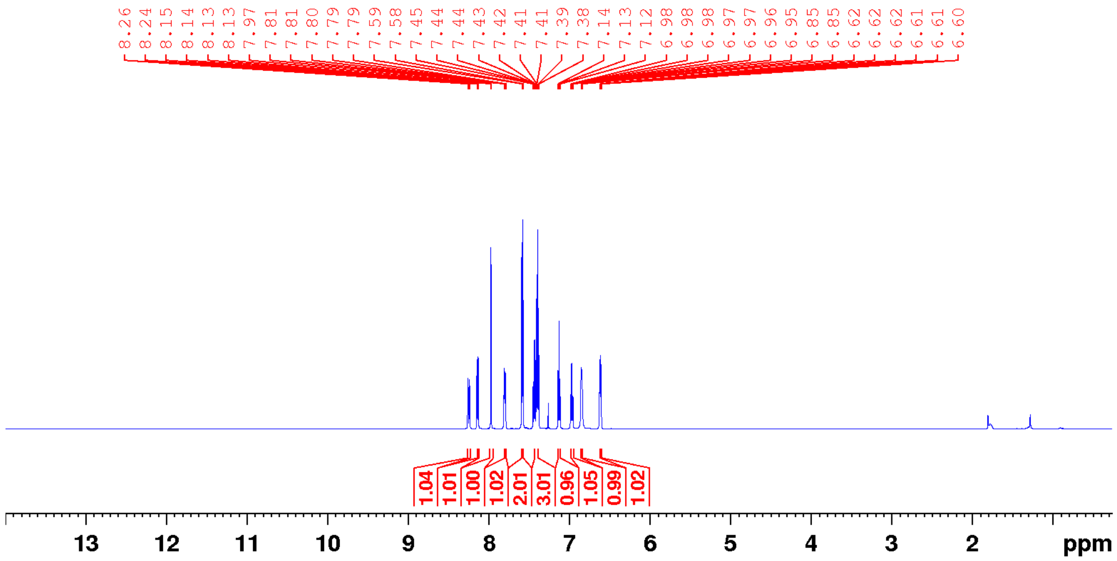
8.26 8.24 8.15 8.14 8.13 8.13 7.97 7.81 7.81 7.80 7.79 7.79 7.59 7.58 7.45 7.44 7.44 7.43 7.42 7.41 7.41 7.39 7.38 7.14 7.13 7.12 6.98 6.98 6.98 6.97 6.97 6.96 6.95 6.85 6.85 6.62 6.62 6.62 6.61 6.61 6.60
1.04 1.01 1.00 1.02 2.01 3.01 0.96 1.05 0.99 1.02
13 12 11 10 9 8 7 6 5 4 3 2 ppm

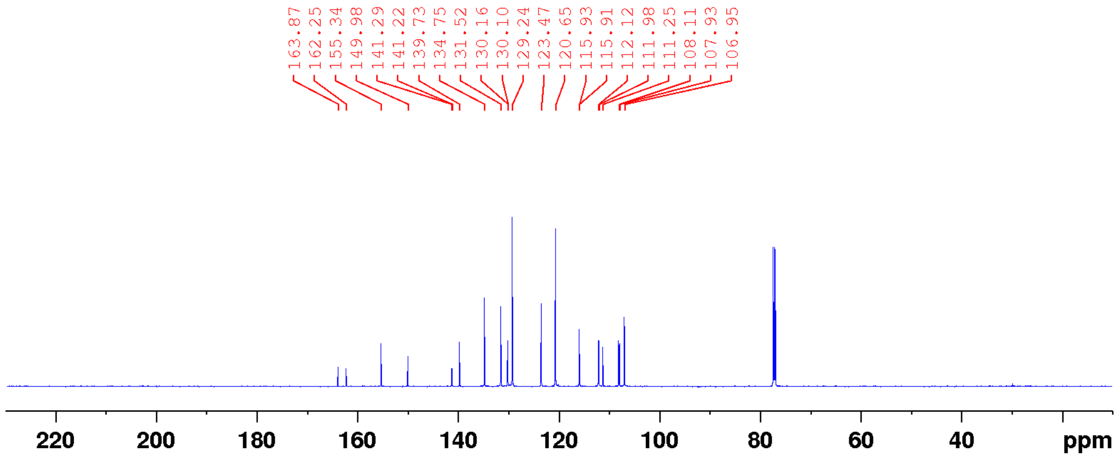
163.87 162.25 155.34 149.98 141.29 141.22 139.73 134.75 131.52 130.16 130.10 129.24 123.47 120.65 115.93 115.91 112.12 111.98 111.25 108.11 107.93 106.95
220 200 180 160 140 120 100 80 60 40 ppm

HPLC of the compounds tested in vivo

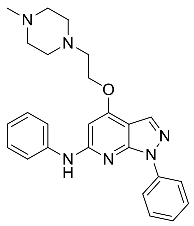

9b

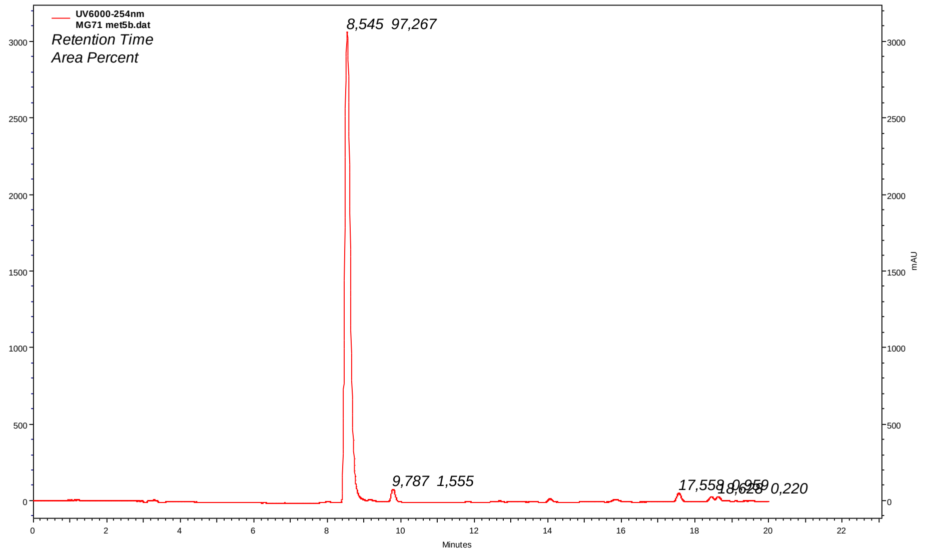

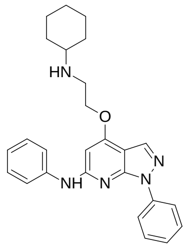

9c

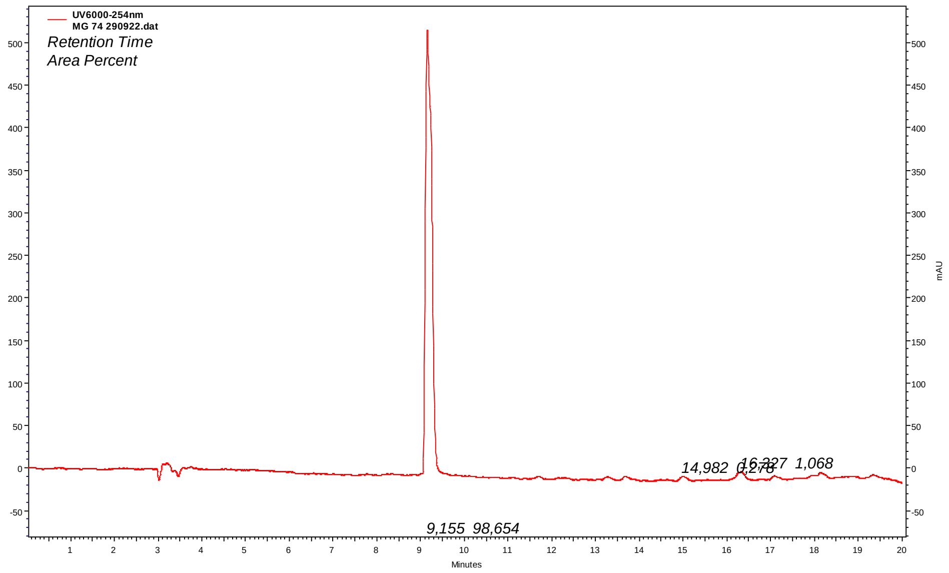

UV6000-254nm
MG 74 290922.dat
Retention Time
Area Percent
9,155 98,654
14,982 0,278
16,327 1,068
Minutes
mAU

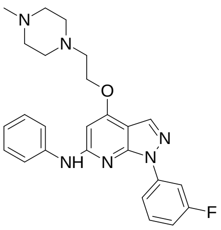

9e

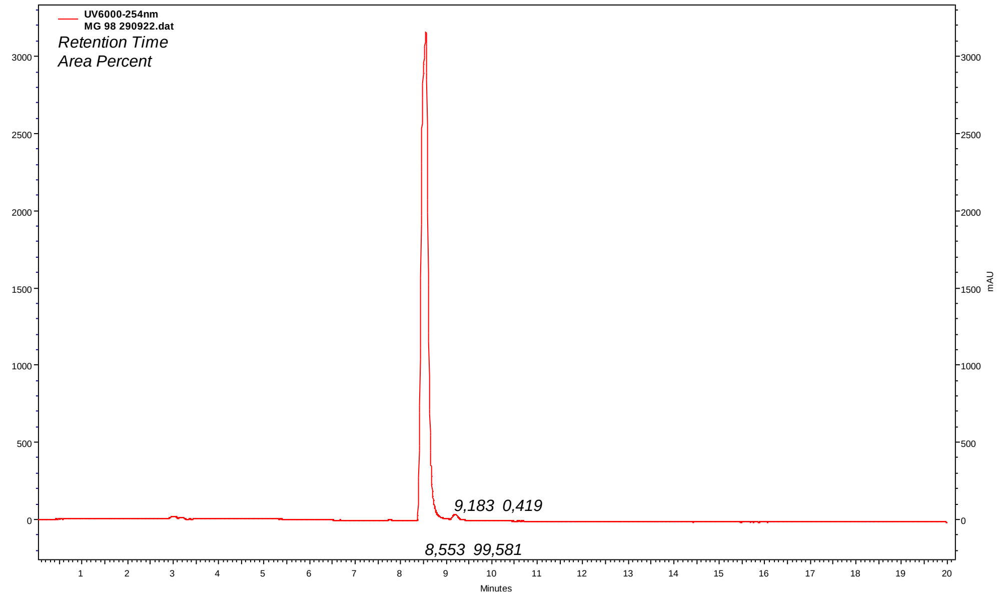

UV6000-254nm
MG 98 290922.dat
Retention Time
Area Percent
9,183 0,419
8,553 99,581
Minutes
mAU
